# Association between Proton Magnetic Resonance Spectroscopy Measurements and CAG Repeat Number in Patients with Spinocerebellar Ataxias 2, 3, or 6

**DOI:** 10.1371/journal.pone.0047479

**Published:** 2012-10-11

**Authors:** Po-Shan Wang, Hung-Chieh Chen, Hsiu-Mei Wu, Jiing-Feng Lirng, Yu-Te Wu, Bing-Wen Soong

**Affiliations:** 1 Institute of Brain Science, National Yang-Ming University, Taipei, Taiwan; 2 Department of Medicine, Municipal Gandau Hospital, Taipei, Taiwan; 3 Department of Neurology, National Yang-Ming University School of Medicine, Taipei, Taiwan; 4 Department of Neurology, Taipei Veterans General Hospital, Taipei, Taiwan; 5 Department of Radiology, Taipei Veterans General Hospital, Taipei, Taiwan; 6 Department of Radiology, National Yang-Ming University School of Medicine, Taipei, Taiwan; 7 Department of Biomedical Imaging and Radiological Sciences, National Yang-Ming University, Taipei, Taiwan; 8 Integrated Brain Research Laboratory, Department of Medical Research and Education, Taipei Veterans General Hospital, Taipei, Taiwan; Pasteur Institute of Lille, France

## Abstract

The aim of this study was to correlate magnetic resonance spectroscopy (MRS) measurements, including that for the N-acetyl aspartate (NAA)/creatine (Cr) ratio in the vermis (denoted V-NAA), right cerebellar hemisphere (R-NAA), and left (L-NAA) cerebellar hemisphere, with the clinical scale for the assessment and rating of ataxia (SARA) score for patients with spinocerebellar ataxia (SCA) types 2, 3, and 6. A total of 24 patients with SCA2, 48 with SCA3, and 16 with SCA6 were recruited; 12 patients with SCA2, 43 with SCA3, and 8 with SCA6 underwent detailed magnetic resonance neuroimaging. Forty-four healthy, age-matched individuals without history of neurologic disease served as control subjects. V-NAA and patient age were used to calculate the predicted age at which a patient with SCA2 or SCA3 would reach an onset V-NAA value. Results showed the following: the NAA/Cr ratio decreased with increasing age in patients with SCA but not in control subjects; the SARA score increased progressively with age and duration of illness; V-NAA showed a better correlation with SARA score than R-NAA in patients with SCA2 or SCA3; the ratio of age to V-NAA correlated well with CAG repeat number; the retrospectively predicted age of onset for SCA2 and SCA3 was consistent with patient-reported age of onset; R-NAA showed a better correlation with SARA score than V-NAA in patients with SCA6; V-NAA and R-NAA correlated with clinical severity (SARA score) in patients with SCA. The correlation between CAG repeat number and age could be expressed as a simple linear function, which might explain previous observations claiming that the greater the CAG repeat number, the earlier the onset of illness and the faster the disease progression. These findings support the use of MRS values to predict age of disease onset and to retrospectively evaluate the actual age of disease onset in SCA.

## Introduction

Spinocerebellar ataxia (SCA) comprises a spectrum of progressive autosomal-dominant neurodegenerative disorders that typically progress over a period of 10 to 20 years, eventually resulting in poor quality of life and a shortened lifespan. One common mutation in patients with SCA1, SCA2, SCA3, SCA6, or SCA17 is an expanded CAG repeat sequence in the human *ATXN1*, *ATXN2*, *ATXN3*, *CACNA1A*, and *TBP* genes, respectively. The length of this CAG repeat expansion has been shown to correlate with the age of disease onset in these patients [1].

SCA3 is the most common form of dominantly inherited ataxia worldwide. In SCA3, the middle cerebellar peduncle and the central white matter of the cerebellum are most affected [1]. The cerebellar cortex and Purkinje neurons are only mildly affected, and the inferior olive is spared [1]. The prevalence of SCA2 is approximately 1 to 2 individuals in 100,000 [1] and is characterized by a marked loss of Purkinje neurons in the cerebellar cortex, as well as a loss of myelinated fibers in the inferior and middle cerebellar peduncles and cerebellar white matter [2]. SCA6 is a polyglutamine disease with a low incidence among Asian populations [3]. Its pathology is characterized mainly by degeneration of Purkinje neurons in the cerebellar cortex, whereas the dentate nucleus, deep cerebellar white matter, and cerebellar peduncles remain unaffected [4], [5].

Magnetic resonance imaging (MRI) and magnetic resonance spectroscopy (MRS) have been used to evaluate the severity of various forms of SCA [6], [7]. In addition, the scale for the assessment and rating of ataxia (SARA), combined with MRI and/or MRS, has been used to evaluate the severity of SCA [6]–[12]. Several chemicals can be analyzed by MRS, and the amino acid N-acetyl aspartate (NAA) has been successfully utilized as a neuronal biomarker for pathologic changes [13], [14]. The aim of the present study was to correlate MRS measurements, including NAA, in patients with SCA2, SCA3, or SCA6 with CAG repeat number and SARA scores, with the goal of establishing a novel method for the prediction of SCA onset and progression, which might help guide the optimization of neuroprotective therapies.

## Materials and Methods

### Patients and Control Subjects

From March 2004 to March 2010, a total of 24 patients with SCA2, 48 with SCA3, and 16 with SCA6 were recruited, and written informed consent was obtained from all. The present study was approved by the Ethics Committee of Taipei Veterans General Hospital and adhered to the principles of the Declaration of Helsinki. All patients underwent periodic neurologic examination and were rated according to SARA [9]. In addition, the duration of disease and the age at examination were recorded. Twelve patients with SCA2, 43 with SCA3, and 8 with SCA6 underwent detailed magnetic resonance neuroimaging studies. Forty-four healthy, age-matched individuals without any history of neurologic disease served as control subjects. The healthy status of the control subjects was confirmed by validating the MRS results according to normal criteria proposed by Danielsen and Ross [10]. Genomic DNA was isolated from peripheral leukocytes, and the length of CAG repeat expansion (repeat number) was determined by polymerase chain reaction, as described previously [15].

### Image Acquisition

**Figure 1:**
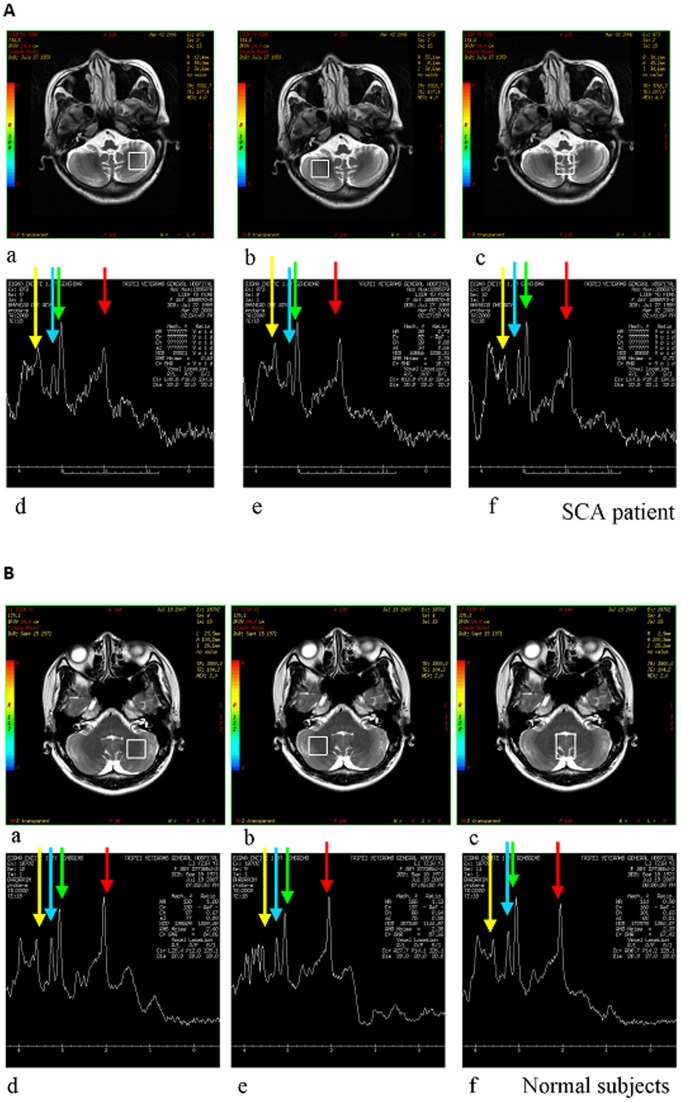
Representative cerebellar proton magnetic resonance (MR) spectra. (A) (Upper left) plane of the left hemisphere in a normal subject at which the MRS signal was acquired; (upper middle) plane of the left hemisphere in a normal subject at which the MRS signal was acquired; (upper right) plane of the vermis in a normal subject at which the MRS signal was acquired; (lower panels) corresponding MR spectra with green arrow (creatine [Cr]), red arrow (N-acetyl aspartate [NAA]), blue arrow (choline [Cho]), and yellow arrow (myoinositol [MI]). (B) (upper left) plane of the left hemisphere in a patient with spinocerebellar ataxia (SCA) at which the MRS signal was acquired; (upper middle) plane of the left hemisphere in a patient with SCA at which the MRS signal was acquired; (upper right) plane of the vermis in a patient with SCA at which the MRS signal was acquired; (lower panels) corresponding MR spectra with green arrow (Cr), red arrow (NAA), blue arrow (Cho), and yellow arrow (MI).

Brain MRI and MRS were performed with a 1.5-T system (Signa EXCITE, GE Medical Systems, Milwaukee, WI, USA). The MRI protocol consisted of an axial, T1-weighted, three-dimensional, fast-spoiled, gradient-recalled acquisition of steady-state images (TR 8.58 msec, TE 3.62 msec, inversion time [TI] 400 msec, slice thickness 1.5 mm) and an axial, T2-weighted, fast spin-echo sequence (TR 4000 msec, TE 256.5 msec, slice thickness 5 mm). After MRI, proton MRS of the bilateral cerebellar hemispheres and the cerebellar vermis was performed with a single-voxel, stimulated echo-acquisition mode sequence (Fig. 1, TR/TE/mixing time/excitations: 3000/15/13.7/96; spectral width  = 2500 Hz, number of points  = 2048, voxel size  = 1.8±2 cm×2 cm ×2 cm). The voxel of interest (VOI) for each subject was selected so as to correspond to the anatomy of the region being investigated (cerebellar hemispheres and vermis) and was performed by the same investigator (JFL) to ensure consistent placement. Individual VOIs were confirmed by two additional investigators (HCC, HMW) from three-dimensional maps overlaid on T2-weighted images for further processing. Measurements were derived by the software associated with the MRI equipment. Care was taken to avoid cerebrospinal fluid–filled spaces within the VOIs. Peak areas for NAA at 2.02 parts per million (ppm), creatine (Cr) at 3.03 ppm, and choline (Cho) at 3.22 ppm were calculated with FuncTool software (GE Healthcare, Milwaukee, WI, USA). Peak integral values were expressed relative to the Cr peak. Metabolite intensity ratios, including NAA/Cr ratios and Cho/Cr ratios, were calculated automatically at the end of each single-voxel acquisition. The NAA/Cho ratio was also calculated for comparison. To ensure high-quality images, MRS results with a full width at half maxima >6 Hz were disqualified from MRS analysis.

### Statistical Analysis

Statistical analyses were performed with SPSS 15.0 statistics software (SPSS Inc., Chicago, IL, USA). Demographic, genetic, and clinical characteristics of the subjects are presented as means with standard deviations, according to SCA type (normal, SCA2, SCA3, and SCA6). These data were also compared by one-way analysis of variance (ANOVA) with a Bonferroni adjustment approach to evaluate the significance of differences between any two SCA types. In addition, the Pearson correlation coefficient was used to assess correlations of CAG repeat number and SARA score with relevant demographic and clinical characteristics. The Spearman correlation coefficient was used for the analysis of left hemisphere NAA (L-NAA) in the control subjects because these data were not normally distributed. Furthermore, a partial correlation analysis of SARA score with MRS measurements was performed by adjusting for age at study (AS), age at onset (AO), disease duration, and CAG repeat number. A standardized regression coefficient was calculated to identify the association between AO and CAG repeat number and the ratio of AO to onset NAA value (AO/onset NAA) by considering the SARA score after adjustment via generalized linear regression analysis. All statistical assessments were considered significant at P<0.05. An adjusted P = 0.01 (0.05/4) was considered significant for the *post hoc* Bonferroni analysis.

## Results

Participant age, disease duration, and AO were not significantly different between groups (Table 1). Values for left and right hemispheric NAA/Cr ratio (L-NAA and R-NAA, respectively), left and right hemispheric Cho/Cr ratio (L-Cho and R-Cho, respectively), vermis NAA/Cr ratio (V-NAA), and vermis Cho/Cr ratio (V-Cho) for healthy control subjects and for the subset of patients who underwent detailed neuroimaging are shown in Table 1. NAA ratios were significantly decreased in both the cerebellar hemispheres and the vermis in all patients with SCA compared to control subjects (P<0.001). In patients with SCA2, Cho values were significantly decreased in the cerebellar hemispheres and the vermis compared to those in healthy subjects (P<0.01). NAA ratios differed among the three groups of patients with SCA (P<0.01). Patients with SCA2 showed lower Cho ratios than did control subjects (P<0.01). In contrast, patients with SCA3 or SCA6 showed no significant differences, with the exception of a lower V-Cho ratio in SCA3 patients (P = 0.025). Among the control subjects, the R-NAA ratio decreased with increasing age (r = –0.352, P<0.05), but the L-NAA and V-NAA ratios exhibited no obvious change with age (Table 2). On the contrary, in patients with SCA3, all NAA ratios decreased with increasing age (Table 2). Therefore, the role of the NAA ratio as a predictor for SCA was examined further.

**Table 1:** Age, CAG repeat number, SARA score, and MRS measurements of study participants.

Variable	Control (n = 44)	SCA2 (n = 24)	SCA3 (n = 48)	SCA6 (n = 16)	*P*
Current age (y)	51.1±18.0	50.8±15.2	48.8±11.4	56.3±9.8	0.116
Age of onset (y)	–	44.3±17.3	40.1±10.5	47.9±8.4	0.075
Disease duration (y)	–	6.5±4.0	8.7±6.2	8.3±7.7	0.838
SARA score	–	10.6±6.4	14.1±8.0	8.3±5.3	0.012*
CAG repeat number	–	40.2±3.5	73.1±4.0	23.5±1.0	<0.001*
MRS measurements:	(n = 44)	(n = 12)	(n = 43)	(n = 8)	
R-NAA	0.99±0.11	0.64±0.12^†††^	0.82±0.14^†††^	0.85±0.09^†††^	<0.001*
R-Cho	0.69±0.09	0.57±0.09††	0.66±0.11	0.70±0.08	0.003*
V-NAA	0.90±0.11	0.67±0.09^†††^	0.79±0.10^†††^	0.78±0.07^†††^	<0.001*
V-Cho	0.68±0.07	0.59±0.07^†††^	0.66±0.07	0.72±0.08	<0.001*
L-NAA	1.00±0.13	0.59±0.18^†††^	0.85±0.14^†††^	0.83±0.13^†††^	<0.001*
L-Cho	0.70±0.09	0.58±0.10††	0.68±0.10	0.70±0.08	0.002*

MRS, magnetic resonance spectroscopy; R-NAA, V-NAA, and L-NAA  = right, vermis, and left hemispheric N-acetyl aspartate/creatine ratio; R-Cho, V-Cho, and L-Cho  = right, vermis, and left hemispheric choline/creatine ratio; SARA, scale for the assessment and rating of ataxia; SCA, spinocerebellar ataxia.

Data are presented as mean ± SD of participants and compared by one-way ANOVA with a Bonferroni adjustment approach.

* P<0.05; significant difference among control, SCA1, SCA3, and SCA6.

†† P<0.01, ^†††^P<0.001; significant difference compared to control.

**Table 2:** Correlation between the current age, onset age, SARA score, CAG repeat number, and MRS measurements for control, SCA2, SCA3, and SCA6, respectively.

	Control		SCA2			SCA3			SCA6	
Variable	Current age	SARA	CAG	Current age	SARA	CAG	Current age	SARA	CAG	Current age
CAG repeats	ND	0.584^**^	NA	NA	0.161	NA	NA	–0.532	NA	NA
Current age	ND	–0.213	–0.842^***^	NA	0.215	–0.615^***^	NA	0.763*	–0.658	NA
Age of onset	ND	–0.346	–0.892^***^	0.977^***^	–0.070	–0.617^***^	0.876^***^	0.203	–0.390	0.654
Duration of illness	ND	0.682^***^	0.650^**^	–0.421	0.510^***^	–0.086	0.410^**^	0.752*	–0.413	0.562
MRS measurements:										
R-NAA	–0.352*	–0.507	–0.524	0.268	–0.553^***^	0.188	–0.572^***^	–0.703	–0.045	–0.597
V-NAA	0.043	–0.819^**^	–0.598*	0.173	–0.748^***^	0.195	–0.364*	–0.047	–0.53	0.269
L-NAA	–0.320^a^	–0.547	–0.810^**^	0.571	–0.536^***^	0.173	–0.566^***^	–0.225	–0.333	–0.269

R-NAA, V-NAA, and L-NAA  = right, vermis, and left hemispheric N-acetyl aspartate/creatine ratio; MRS, magnetic resonance spectroscopy; ND  = not derived; NA  = not assessed; SARA, scale for the assessment and rating of ataxia; SCA, spinocerebellar ataxia.

Data are shown with Pearson correlation coefficients (r). ^a^Spearman correlation was applied, owing to the fact that L-NAA in the control group was not normally distributed.

* P<0.05, ^**^P<0.01, ^***^P<0.001; significant correlation.

With respect to CAG repeats, the number of CAG repeats correlated negatively with AO in patients with SCA2 or SCA3 (Table 2). The correlation among patients with SCA6 was not significant, which could have been due to the limited sample size (n = 8). Among patients with SCA2, SCA3, or SCA6, the SARA score increased with the duration of illness (Table 2). NAA ratios correlated with SARA scores with various correlation coefficients in patients with SCA2 or SCA3. In patients with SCA3, R-NAA and L-NAA ratios showed similar correlation coefficients (R-NAA, r = –0.553, P<0.001; L-NAA, r = –0.536, P<0.001). Therefore, for subsequent analyses, the R-NAA ratio was used to represent hemispheric NAA. In addition, the V-NAA ratio was also negatively correlated with SARA score in patients with SCA3 (r = –0.748, P<0.001). In patients with SCA2, the correlation was significant for SARA score with V-NAA ratio but not with R-NAA ratio or L-NAA ratio (V-NAA, r = –0.819, P<0.01; R-NAA, r = –0.507, P = 0.141; L-NAA, r = –0.547, P = 0.065). Patients with SCA6 showed negative correlations between SARA score and NAA ratios; however, these did not reach statistical significance (all P>0.05) (Table 2). Detailed regression analysis of the lower ranges of the SARA scores (near the intercept) showed that at disease onset (SARA  = 0), patients with SCA had V-NAA values of 0.7866 (SCA2), 0.8576 (SCA3), and 0.7877 (SCA6) (data not shown).

With the progression of SCA, NAA decreased, and the AO/onset NAA ratio increased. We found that the CAG repeat number was negatively associated with the AO/onset NAA ratio (Table 3). In patients with SCA2 or SCA3, the increased standardized regression coefficients represented a negative association between CAG repeat number and the AO/onset NAA ratio, which might have been increased because the SARA score was adjusted for either SCA2 or SCA3. There were no significant differences in patients with SCA6 (Table 3).

**Table 3:** Association of age of onset and CAG repeat number with the ratio of age of onset to onset NAA value.

		SCA2 Group		SCA3 Group		SCA6 Group	
Dependent variable	Predictor	Without adjusted SARA	With adjusted SARA	Without adjusted SARA	With adjusted SARA	Without adjusted SARA	With adjusted SARA
Onset age	Onset age/onset R-NAA	0.751^**^	0.820^**^	0.577^***^	0.644^***^	0.617	0.780*
Onset age	Onset age/onset V-NAA	0.830^***^	0.952^***^	0.665^***^	0.815^***^	0.566	0.658
CAG repeats	Onset age	–0.892^***^	–0.906^***^	–0.617^***^	–0.633^***^	–0.390	–0.341
CAG repeats	Onset age/onset R-NAA	–0.561	–0.728*	–0.395^**^	–0.459^**^	–0.492	–0.128
CAG repeats	Onset age/onset V-NAA	–0.582*	–0.835^**^	–0.540^***^	–0.717^***^	–0.365	0.085

R-NAA and V-NAA  = right and vermis hemispheric N-acetyl aspartate/creatine ratio; SARA, scale for the assessment and rating of ataxia; SCA, spinocerebellar ataxia.

Results are shown as standardized regression coefficients via regression analysis with or without adjusted SARA score. Greater absolute value of standardized regression coefficients indicates greater association between dependent variable and predictor.

* P<0.05; ^**^P<0.01; ^***^P<0.001; significance of standardized regression coefficients.

Consistent with the above analyses, the AO/onset V-NAA ratio correlated with AO in patients with SCA3 (r = 0.665, P<0.001, Table 3). Of note, the measurements were from patients with different SARA scores. When the patient's SARA score was controlled for during the correlation analysis, the coefficient increased to 0.815 (P<0.001). A correlation of the AO/onset R-NAA ratio with AO was also observed in patients with SCA2 or SCA6, with the exception of patients with SCA6 with adjusted SARA scores (Table 3). With SARA score adjustment, the correlation of AO/onset NAA ratio with AO and with CAG repeat number was more obvious and significant compared to without adjustment.

**Figure 2:**
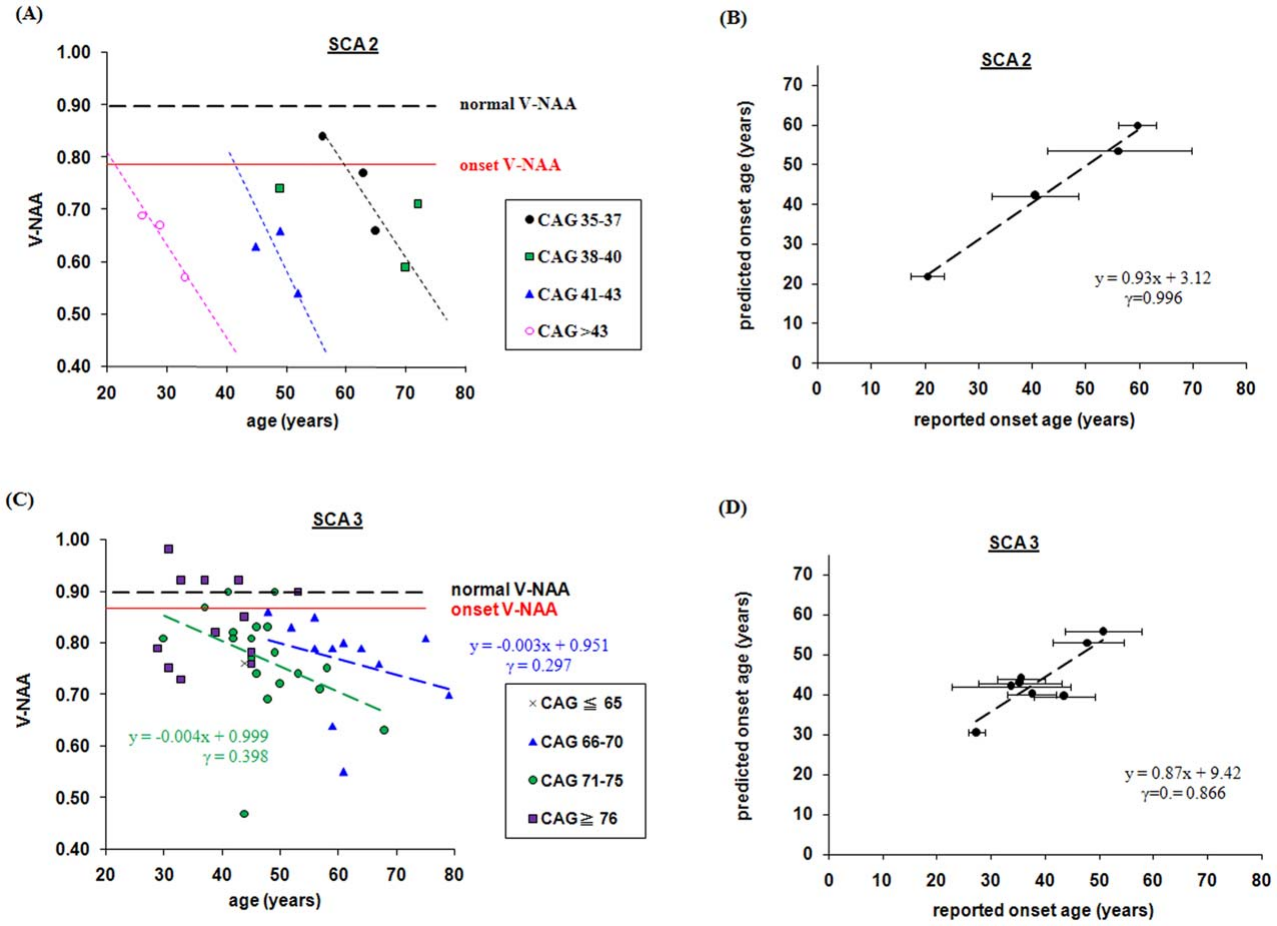
Changes in N-acetyl aspartate/creatine ratio (NAA) with age and CAG repeat number. Changes in NAA ratio with age were related to the number of CAG repeats in patients with spinocerebellar ataxia 2 (SCA2) (A) or SCA3 (C). In A and C, the onset vermis NAA value (V-NAA) was calculated based on the correlation of the scale for the assessment and rating of ataxia (SARA) score and V-NAA and was used to retrospectively predict the age of onset of for CAG repeat group. The predicted age of onset and the average reported age of onset are plotted in B (SCA2) and D (SCA3). The error bars denote the standard deviation of reported ages.

We also found that patients with SCA exhibited differences in CAG repeat number. When they were grouped based on CAG repeat number, the decrease of NAA ratio with age was more obvious (Fig. 2A, C). Overall, the greater the CAG repeat number, the earlier the decrease occurred, especially in patients with SCA2. Notably, in patients with SCA3, the greater the CAG repeat number, the faster the disease progression. When two or more NAA ratios (at different time points) were available, the change in NAA ratio could be used to predict the rate of disease progression. To test this, data from patients with similar CAG repeat numbers were used to predict age of disease onset. The V-NAA and AS of these patients were used to calculate the age at which the patient had a predicted onset V-NAA. As mentioned above, at the onset of SCA2 or SCA3, the V-NAA values were 0.7866 and 0.8576, respectively. The calculated age was considered the retrospectively predicted AO of the patient with the corresponding CAG repeat number. Interestingly, the average reported AO showed a good correlation with the predicted AO (Fig. 2B, SCA2, r = 0.996, P<0.001; Fig. 2D, SCA3, r = 0.866, P<0.001).

## Discussion

In the present study, we observed a good correlation between MRS measurement of the NAA/Cr ratio and SARA score. The NAA/Cr ratio decreased with increasing age among patients with SCA. This decrease occurred earlier with increased CAG repeat number, and AO/NAA ratio correlated well with CAG repeat number and disease AO. These findings suggest that the rate of change of the NAA ratio can be used to follow the progression of SCA and might also be used to predict the AO of the disease.

### MRS Measurement and SARA Score

SARA score has been used to monitor the clinical progression of SCA [9]. However, SARA is a clinical rating scale, and different doctors might rate the same patient slightly differently. In addition, like other neurodegenerative diseases, SCA shows a great deal of variation in clinical presentation. Hence, SARA score might not faithfully reflect absolute longitudinal disease progression. MRS is an objective biochemical measurement of neuronal viability, which is not influenced by the observer [16]. Measurements of cerebellar NAA levels by proton MRS have been shown to faithfully distinguish patients with SCA1 from healthy subjects, with very high specificity and sensitivity [8]. Thus, MRS measurements such as NAA ratios can be utilized to noninvasively monitor the neuronal and glial status of patients with ataxia.

In polyglutamine (polyQ) diseases, when the polyQ tract (coded for by CAG repeats) reaches the pathologic threshold, the protein becomes neurotoxic. Consequently, this leads to selective neuronal cell death [17], [18] along with neurochemical alterations detected by MRS including decreases in acetylcholine and NAA [6], [8], [12], [19]. NAA has long been used as a marker of neuronal integrity and viability and is one of the most important brain metabolites detected by proton MRS [12]. Numerous studies have demonstrated a correlation between NAA level and SCA progression, but none of these reports has established an association with AO. Compared to control subjects, patients with SCA1 or SCA2 show a decrease in NAA in the pons and cerebellar hemisphere [13]. Previous studies have also demonstrated significant decreases of NAA and choline in patients with multiple system atrophy with cerebellar features (MSA-C) or SCA2 [6], [7], as well as in patients with SCA1 [20]. The decrease in NAA we observed in the cerebellum of patients with SCA2, SCA3, or SCA6 is consistent with the results of these studies and suggests that NAA is a good marker for SCA.

We measured NAA ratios in the vermis and both cerebellar hemispheres. V-NAA showed a better correlation with SARA score and CAG repeat number than R-NAA in patients with SCA2 or SCA3. In contrast, R-NAA showed a better correlation in patients with SCA6. It is known that change in NAA is related to neuronal viability. The pathologic changes of SCA2 indicate a marked loss of Purkinje neurons in the cerebellar cortex, as well as a loss of myelinated fibers, with gliosis in the inferior and middle cerebellar peduncles, cerebellar white matter, and fasciculus cuneatus [2], [21]. In SCA3, the cerebellar cortex and Purkinje neurons are only mildly affected [22]. Instead, the middle cerebellar peduncle and central white matter of the cerebellum are involved [22]. In SCA6, the pathology involves mainly degeneration of Purkinje neurons in the cerebellar cortex, leaving the dentate nucleus, deep cerebellar white matter, and cerebellar peduncles unaffected [4], [5]. Our prior fluorodeoxyglucose positron emission tomography study also demonstrated that the pattern of cerebellar glucose hypometabolism also differs in SCA2, SCA3, and SCA6 [23]. The glucose metabolic rate of the entire cerebellum is low in patients with SCA2, whereas it is low in the central cerebellum in patients with SCA3 and in the cerebellar cortex in patients with SCA6 [23]. In the present study, the stronger correlations between V-NAA and SARA score and between AS/V-NAA and CAG repeat number or AO in patients with SCA2 or SCA3 suggest that the neuropathy involves mainly the central portion of the cerebellum such as the vermis, dentate nucleus, or cerebellar white matter. Although the patient numbers were limited, we nonetheless observed a better correlation between R-NAA and SARA score than between V-NAA and SARA score, suggesting more severe neuropathy in the cerebellar cortex of patients with SCA6. Despite the fact that we could not confirm these results with histopathologic changes in our patients, these findings are consistent with previously described pathologic changes in patients with SCA2, SCA3, or SCA6 [1]–[5].

### Decreased NAA, CAG Repeat Number, and Disease Onset

It is well known that AO correlates negatively with CAG repeat number. In the presence of increased CAG repeats, the process of neuropathologic injury is expedited [24] resulting in an early disease onset. Results of the present study demonstrated that the AO for patients with SCA3 or SCA2 correlated with the number of CAG repeats. We found that the decrease in cerebellar NAA ratio started before the onset of disease. Importantly, we found a negative correlation between AO/NAA ratio and CAG repeats. With the progression of SCA, NAA ratio decreased, and the age/NAA ratio increased. We also found that the onset V-NAA value was similar or the same among patients with SCA2 or SCA3. Therefore, it is possible that at the so-called AO, cerebellar viability may be reduced to a critical point such that the residual cerebellar neurons can no longer compensate for the decreased function owing to the loss of neurons. Use of the NAA ratio might allow for a more accurate estimation of this critical point, which might precede the patient's disease AO, when they begin to suffer from physical symptoms such as ataxia. Specifically, our results suggest that the correlation between CAG repeat number and AO might be explained by the correlation between CAG repeat number and AO/NAA ratio. From the equation we derived for Table 3, the more CAG repeats a patient has, the lower the AO and the lower the value of the AO/onset NAA ratio. Given that the onset V-NAA value was similar or the same among patients with SCA3 or SCA2, we may conclude that the greater the CAG repeat number, the earlier the patient will experience ataxia. In addition, the patients in the present study had various SARA scores, and the increase in the correlation coefficient and statistical significance after controlling for SARA score supports the rationale and validity of our model (Table 3). However, these findings should be further validated with larger sample sizes and measurements during the various stages of SCA development.

### Predictive Value of NAA Ratio for the Progression of SCA

Because the NAA ratio was closely correlated with the SARA score, measurements of NAA could be used to predict the progression of SCA even when the CAG repeat number is controlled for. We assumed that at disease onset, the SARA score is zero. Detailed regression analysis suggested that the values of V-NAA at the onset of SCA2 and SCA3 were 0.7866 and 0.8576, respectively. We grouped the patients based on CAG repeat number and retrospectively predicted AO. The resulting predicted AO fit well with the reported AO. As discussed above, for patients with SCA6, cerebellar hemispheric NAA should be used instead of V-NAA. Interestingly, as shown in Fig. 2, cerebellar NAA decreased linearly with patient age. With longitudinal measurements of NAA on the same presymptomatic subject, we should also be able to predict when the NAA level will reach the critical point and the presymptomatic subject will started experiencing ataxia.

CAG repeat size has a major effect on phenotype expression and affects the frequency of several of the clinical signs [24]. Large and very large CAG repeat numbers lead to extracerebellar symptoms [24]. The occurrence of SCA3 at a younger age might not be accompanied by a decrease in NAA, such as in those with more than 71 CAG repeats (Fig. 2C). In the present study, we did not exclude these values from analysis, and all of the MRS measurements were performed after SCA onset. Further work with preclinical measurements and larger sample sizes should be performed to confirm the effectiveness of our prediction model and refine its scope.

A similar study of a large cohort of patients with Huntington disease (2913 individuals from 40 centers worldwide) successfully developed a parametric survival model based on CAG repeat number to predict the probability of AO for Huntington disease (based on motor neurologic symptoms rather than psychiatric symptoms) at different ages for individual patients [25]. However, ours is the first study to date to suggest that NAA measurements can be used for such a prediction. This finding should allow for novel predictive analyses based on repeated measurements from a patient even without CAG repeat number information. Furthermore, since NAA ratios correlate with SARA score, NAA ratios should be used to predict the severity of SCA in the future or be used as an indicator of the patient's current severity. This longitudinal measurement will be particularly useful in screening for SCA in a high-risk population (*e.g.* relatives of patients with SCA). Thus, when NAA decreases are observed, further genetic tests and clinical examinations should be performed. This notion is consistent with the hypothesis that MRS biomarkers, such as NAA, are sensitive to the early and progressing states of ataxia disorders, which might even include stages of the disease before the development of major pathologic changes and ataxia [11]. Currently, there are no clinically available treatments for SCA [26]. We hope that in the future, an early diagnosis will allow for the reversal of motor dysfunction in diseases such as SCA1 by suppressing the expression of mutant *ATXN1* and thereby preventing further neurodegeneration [27], [28].

### Potential Study Limitations

There are several potential limitations of the present study. First, the self-reported AO is not always as precise as we would wish. This is very common in retrospective studies and is difficult to totally avoid. Second, the sample size was limited, especially for SCA2 and SCA6, which is particularly important considering that NAA/Cr measurements have an inherent error rate of 10% to 15%. The relatively small sample size could very well introduce variability. Third, given that the software associated with the MRI system derived measurements, we were not able to assess interobserver agreement. Fourth, measurements before SCA onset would validate the power of our predictions. In addition, we would have liked to obtain an extensive neurochemical profile (e.g. ascorbate, gama-aminobutyric acid, Gln, Glu, tCho, tau, etc.). However, this requires a high-end 3T MRS system, which we do not have access for the study. Despite these limitations, we managed to construct a model based on MRS measurements to predict the AO and progression of SCAs, which could help guide the optimization of future neuroprotective therapies.
